# Scrutinizing joint remote state preparation under decoherence

**DOI:** 10.1038/s41598-023-34949-4

**Published:** 2023-05-18

**Authors:** Cookey Iyen, Babatunde James Falaye, Muhammad Sanusi Liman

**Affiliations:** 1 Department of Physics, Federal University of Lafia, Lafia, 950101 Nasarawa State Nigeria; 2 Department of Pure and Applied Physics, Federal University Wukari, Wukari, 670101 Taraba State Nigeria

**Keywords:** Information theory and computation, Quantum information, Quantum mechanics

## Abstract

This research examines the effect of an open system containing the squeezed generalized amplitude damping channel on the joint remote preparation quantum communication protocol using a maximally entangled two-qubit state. Our findings indicate that the fidelity of a quantum system in contact with a non-zero temperature thermal bath can be enhanced by varying the squeezing parameters. These parameters include the squeezing phase of the channel $\Phi$ and the amount of squeezing of the channel *r*.

## Introduction

One of the most amazing aspects of quantum physics is the quantum entanglement (QE)^1,2^. QE is a phenomenon in which particles produced at the same source appear to be linked irrespective of their distance apart, in the sense that whatever is done to one of the particles affects the quantum state of other member(s) of the entangled particles. QE is crucial in modern quantum information processing^3^. Many quantum communication architectures have used QE, including quantum teleportation (QT)^4–10^ and remote state preparation (RSP)^11,12^.

While QT, as proposed by Bennett et al.^13^, sends an unknown state to a remote receiver via a previously established entanglement channel, a new protocol known as Remote State Preparation (RSP) has been developed^14,15^, in which the sender knows the state to be transmitted but the receiver does not. Even-though the cost of classical information transmission and that for quantum information transmission are almost the same when using the QT protocol, it has been observed that it is possible to obtain some cost advantages when the RSP-based protocol is used. This advantage has made RSP a very important quantum communication protocol. Other RSP-based protocols, such as optimal remote state preparation^16^, low entanglement remote state preparation^17^, joint remote state preparation^12,18^, multiparty remote state preparation^19^, oblivious remote state preparation^20^, and others, have recently made significant advances.

RSP has been experimented with in the laboratory by using liquid-state nuclear magnetic resonance to transmit a qubit from a hydrogen to a carbon nucleus, by using photonic quantum systems and by propagating two-mode squeezed microwave states^21–23^. Many theoretical works on the RSP protocol have also been published; see, for example, Refs.^21,24–26^. In the Joint Remote State Preparation (JRSP)^27,28^, the initial state to be sent to a remote receiver is partially shared among multiple senders that are located at different locations. In this protocol, none of the senders has the full information; however, the receiver is able to recreate the initial state of the information received by performing certain unitary transformations on the received state.

Despite the fact that much research has been conducted on JRSP protocols without focusing on the effect of environmental noise on the protocol^12,28,29^, however, from a more realistic perspective, it is clear that it is not possible for a quantum state not to interact with and be affected by its environment. A system that does not interact with or is not influenced by its environment is called a closed system, while a system that interacts with and is affected by its environment is called an open system^30–34^. The effect of this channel on transmitted information affects the quality of information that reaches the receiver from the sender. The effect of environmental noise such as external magnetic field and phase damping on the entanglement of real physical systems have been widely researched^35–38^ and appreciable research has been executed on a number of noise models that attempt to simulate the effect of different channel parameters on transmitted information.

Adepoju et al.^39^, for example, considered bit-flip channel, phase-flip channel, bit-phase-flip channel, amplitude damping channel, phase damping channel, and depolarizing channel when studying the JRSP of a two-qubit equatorial state in a quantum noisy channel. Guan et al.^3^ investigated the JRSP of an arbitrary 2-qubit state in both amplitude damping (AD) and phase damping (PD) channels, and a number of other authors^40–44^ have investigated JRSP in various noisy channels. However, to the best of our knowledge, no research has been carried out on a JRSP in the Squeezed Generalized Amplitude Damping (SGAD) channel.

We recognized that a lot of research has already been carried out on the generalized amplitude damping channel but it will be of interest to know how JRSP of quantum communication protocol is affected when subjected to both amplitude damping and squeezing noise. In this research, we consider the effect of the SGAD channel on the fidelity of information transmitted in the JRSP protocol of an arbitrary two-qubit state. The JRSP protocol has been extensively explained by other authors; our aim in this work is mainly to determine the effect of SGAD on the already established JRSP model of an arbitrary 2-qubit state. For quantum systems that have interactions with their environment, the origin of the noise is the decoherence effect, which is brought about by the system’s interaction with the channel.

The advancement over time of a quantum system that is continuously influenced by its environment can be defined using the master’s equation in the Lindblad form^45^ for the density operator $\rho (t)$^3,46,47^ within the framework of Markov and Born approximations. Also in JRSP architecture, the senders and receivers are required to share a quantum state with one another as a communication channel to finalize the preparation. In a practical JRSP, the channel state must be generated by one of the parties that make up the JRSP, and the resulting qubits must be shared with the respective members through a noisy channel. This procedure changes the pure channel state into a mixed state.

In this research, Kraus operators are employed to give a representation of the consequences of the noisy channel. One major type of noise is considered; the squeezed generalized amplitude damping noise, which is a generalized type of noise that may be used to model the amplitude damping (AD) and the generalized amplitude damping (GAD) noises by assigning particular values to some of their parameters. Fidelity was used to compare the closeness of the final state received by the receiver to the initial state sent by the sender and was also used to quantify how much information was lost in the JRSP process. Finally, the results are discussed and comparisons are made between the AD, GAD, and SGAD channels, and we are able to show how each of the parameters of the SGAD influenced the fidelity of the JRSP system.

This paper is arranged as follows: In Section “Review of joint remote preparation of two qubit system”, we explore the JRSP of a two-qubit system. In Section “Fidelity computation procedure”, we introduce the fidelity computation framework. In Section “Kraus operators, fidelity computation of SGAD channel and associated dissipative channels”, we compute the fidelities of the SGAD channel and other associated dissipative channels, and in Section “Discussion and conclusion”, we discuss our results and conclude.

## Review of joint remote preparation of two qubit system

In this work, we use the Joint Remote State Preparation as introduced by Wang et al.^48^. They used a six-qubit cluster state to prepare an arbitrary two-qubit state. Their scheme is as follows: If we consider two participants named Alice and Bob who are interested in assisting a third remote participant named Caleb, they prepare an arbitrary two-qubit quantum state, which may be described as (1)

1 $$\begin{aligned} \vert {B}\rangle =a_0e^{i\theta _0}\vert {00}\rangle +a_1e^{i\theta _1}\vert {01}\rangle +a_2e^{i\theta _2}\vert {10}\rangle +a_3e^{i\theta _3}\vert {11}\rangle , \end{aligned}$$

where coefficients $a_i(i=0,1,2,3)$ in (1) are real and satisfy the normalization condition $a_0^2 + a_1^2+a_2^2+a_3^2=1$, as well as $\theta _j \in [0,2\pi ](j=0,1,2,3)$. The information in $\vert {B}\rangle$ is shared between Alice and Bob; Alice holds the amplitude information $a_i$ and Bob holds on to the phase information $\theta _j$. To be able to send the original state to Caleb, they require a quantum state as a communication channel, such as (2)

2 $$\begin{aligned} \vert {CH}\rangle =\frac{1}{2}(\vert {000000}\rangle +\vert {000111}\rangle +\vert {111000}\rangle +\vert {111111}\rangle )_{123456}, \end{aligned}$$

where Alice’s particles are (1, 4), Bob’s (2, 5), and Caleb’s (3, 6). Alice and Bob choose an orthogonal basis to measure their local qubits. The forms of the measurement bases are as follows: we have $\vert {\rho ^1}\rangle _{14}$,$\vert {\rho ^2}\rangle _{14}$,$\vert {\rho ^3}\rangle _{14}$, $\vert {\rho ^4}\rangle _{14}$. Her local measurement basis is as follows;

3 $$\begin{aligned} \vert {\rho ^1}\rangle _{14}=a_0\vert {00}\rangle +a_1\vert {01}\rangle +a_2\vert {10}\rangle -a_3\vert {11}\rangle ,\nonumber \\ \vert {\rho ^2}\rangle _{14}=a_1\vert {00}\rangle -a_0\vert {01}\rangle -a_3\vert {10}\rangle -a_2\vert {11}\rangle , \nonumber \\ \vert {\rho ^3}\rangle _{14}=a_2\vert {00}\rangle +a_3\vert {01}\rangle -a_0\vert {10}\rangle +a_1\vert {11}\rangle , \nonumber \\ \vert {\rho ^4}\rangle _{14}=a_3\vert {00}\rangle -a_2\vert {01}\rangle +a_1\vert {10}\rangle +a_0\vert {11}\rangle . \end{aligned}$$

We have $\vert {\eta ^1}\rangle _{25}$, $\vert {\eta ^2}\rangle _{25}$, $\vert {\eta ^3}\rangle _{25}$, $\vert {\eta ^4}\rangle _{25}$, for Bob, and his local measurement basis is:

4 $$\begin{aligned} \vert {\eta ^1}\rangle _{25}=\frac{1}{2}(e^{-i\theta _0}\vert {00}\rangle +e^{-i\theta _1}\vert {01}\rangle +e^{-i\theta _2}\vert {10}\rangle +e^{-i\theta _3}\vert {11}\rangle ,\nonumber \\ \vert {\eta ^2}\rangle _{25}=\frac{1}{2}(e^{-i\theta _0}\vert {00}\rangle -e^{-i\theta _1}\vert {01}\rangle +e^{-i\theta _2}\vert {10}\rangle -e^{-i\theta _3}\vert {11}\rangle ,\nonumber \\ \vert {\eta ^3}\rangle _{25}=\frac{1}{2}(e^{-i\theta _0}\vert {00}\rangle -e^{-i\theta _1}\vert {01}\rangle -e^{-i\theta _2}\vert {10}\rangle +e^{-i\theta _3}\vert {11}\rangle ,\nonumber \\ \vert {\eta ^4}\rangle _{25}=\frac{1}{2}(e^{-i\theta _0}\vert {00}\rangle +e^{-i\theta _1}\vert {01}\rangle -e^{-i\theta _2}\vert {10}\rangle -e^{-i\theta _3}\vert {11}\rangle . \end{aligned}$$

Considering Eqs. (3) and (4), it implies that $\vert {CH}\rangle$ can be written as:

5 $$\begin{aligned} {\left\{ \begin{array}{ll} \vert {CH}\rangle =\frac{1}{4}\vert {\rho ^1}\rangle _{14}[\vert {\eta ^1}\rangle _{25}(a_0e^{i\theta _0}\vert {00}\rangle +a_1e^{i\theta _1}\vert {01}\rangle +a_2e^{i\theta _2}\vert {10}\rangle +a_3e^{i\theta _3}\vert {11}\rangle )_{36} \\ + \, \vert {\eta ^2}\rangle _{25}(a_0e^{i\theta _0}\vert {00}\rangle -a_1e^{i\theta _1}\vert {01}\rangle +a_2e^{i\theta _2}\vert {10}\rangle -a_3e^{i\theta _3}\vert {11}\rangle )_{36}\\ +\vert {\eta ^3}\rangle _{25}(a_0e^{i\theta _0}\vert {00}\rangle -a_1e^{i\theta _1}\vert {01}\rangle -a_2e^{i\theta _2}\vert {10}\rangle +a_3e^{i\theta _3}\vert {11}\rangle )_{36} \\ + \, \vert {\eta ^4}\rangle _{25}(a_0e^{i\theta _0}\vert {00}\rangle +a_1e^{i\theta _1}\vert {01}\rangle -a_2e^{i\theta _2}\vert {10}\rangle -a_3e^{i\theta _3}\vert {11}\rangle )_{36})]\\ +\frac{1}{4}\vert {\rho ^2}\rangle _{14}[\vert {\eta ^1}\rangle _{25}(a_1e^{i\theta _0}\vert {00}\rangle -a_0e^{i\theta _1}\vert {01}\rangle -a_3e^{i\theta _2}\vert {10}\rangle +a_2e^{i\theta _3}\vert {11}\rangle )_{36} \\ + \, \vert {\eta ^2}\rangle _{25}(a_1e^{i\theta _0}\vert {00}\rangle +a_0e^{i\theta _1}\vert {01}\rangle -a_3e^{i\theta _2}\vert {10}\rangle -a_2e^{i\theta _3}\vert {11}\rangle )_{36} \\ +\vert {\eta ^3}\rangle _{25}(a_1e^{i\theta _0}\vert {00}\rangle +a_0e^{i\theta _1}\vert {01}\rangle +a_3e^{i\theta _2}\vert {10}\rangle +a_2e^{i\theta _3}\vert {11}\rangle )_{36} \\ + \, \vert {\eta ^4}\rangle _{25}(a_1e^{i\theta _0}\vert {00}\rangle -a_0e^{i\theta _1}\vert {01}\rangle +a_3e^{i\theta _2}\vert {10}\rangle -a_2e^{i\theta _3}\vert {11}\rangle )_{36})] \\ + \, \frac{1}{4}\vert {\rho ^3}\rangle _{14}[\vert {\eta ^1}\rangle _{25}(a_2e^{i\theta _0}\vert {00}\rangle +a_3e^{i\theta _1}\vert {01}\rangle -a_0e^{i\theta _2}\vert {10}\rangle -a_1e^{i\theta _3}\vert {11}\rangle )_{36} \\ +\vert {\eta ^2}\rangle _{25}(a_2e^{i\theta _0}\vert {00}\rangle -a_3e^{i\theta _1}\vert {01}\rangle -a_0e^{i\theta _2}\vert {10}\rangle +a_1e^{i\theta _3}\vert {11}\rangle )_{36} \\ + \, \vert {\eta ^3}\rangle _{25}(a_2e^{i\theta _0}\vert {00}\rangle -a_3e^{i\theta _1}\vert {01}\rangle +a_0e^{i\theta _2}\vert {10}\rangle -a_1e^{i\theta _3}\vert {11}\rangle )_{36} \\ + \, \vert {\eta ^4}\rangle _{25}(a_2e^{i\theta _0}\vert {00}\rangle +a_3e^{i\theta _1}\vert {01}\rangle +a_0e^{i\theta _2}\vert {10}\rangle +a_1e^{i\theta _3}\vert {11}\rangle )_{36}] \\ + \, \frac{1}{4}\vert {\rho ^4}\rangle _{14}[\vert {\eta ^1}\rangle _{25}(a_3e^{i\theta _0}\vert {00}\rangle -a_2e^{i\theta _1}\vert {01}\rangle +a_1e^{i\theta _2}\vert {10}\rangle -a_0e^{i\theta _3}\vert {11}\rangle )_{36} \\ + \, \vert {\eta ^2}\rangle _{25}(a_3e^{i\theta _0}\vert {00}\rangle +a_2e^{i\theta _1}\vert {01}\rangle +a_1e^{i\theta _2}\vert {10}\rangle +a_0e^{i\theta _3}\vert {11}\rangle )_{36} \\ + \, \vert {\eta ^3}\rangle _{25}(a_3e^{i\theta _0}\vert {00}\rangle +a_2e^{i\theta _1}\vert {01}\rangle -a_1e^{i\theta _2}\vert {10}\rangle -a_0e^{i\theta _3}\vert {11}\rangle )_{36} \\ + \, \vert {\eta ^4}\rangle _{25}(a_3e^{i\theta _0}\vert {00}\rangle -a_2e^{i\theta _1}\vert {01}\rangle -a_1e^{i\theta _2}\vert {10}\rangle +a_0e^{i\theta _3}\vert {11}\rangle )_{36}]. \end{array}\right. } \end{aligned}$$

Caleb will only be able to obtain the original message if Alice’s measurements are $\vert {\rho ^1}\rangle _{14}$, as he can easily obtain the initial message by performing simple unitary operations on the message he receives, according to Eq. (5). The summaries of Alice’s measurements, Bob’s measurements, and Caleb’s measurements for these four conditions are summarized in Table 1.

**Table 1:** Local measurements made by Alice, Bob, and Caleb and the transformation made by Caleb to obtain the original message.

Alice’s measurement outcome	Bob’s measurement outcome	Caleb’s measurement outcome	Required transformation	Outcome after transformation
$\vert {\rho ^1}\rangle _{14}$	$\vert {\eta ^1}\rangle _{25}$	$a_0e^{i\theta _0}\vert {00}\rangle +a_1e^{i\theta _1}\vert {01}\rangle +a_2e^{i\theta _2}\vert {10}\rangle +a_3e^{i\theta _3}\vert {11}\rangle$	$\mathbb {1} \otimes \mathbb {1}$	$a_0e^{i\theta _0}\vert {00}\rangle +a_1e^{i\theta _1}\vert {01}\rangle +a_2e^{i\theta _2}\vert {10}\rangle +a_3e^{i\theta _3}\vert {11}\rangle$
$\vert {\rho ^1}\rangle _{14}$	$\vert {\eta ^2}\rangle _{25}$	$a_0e^{i\theta _0}\vert {00}\rangle -a_1e^{i\theta _1}\vert {01}\rangle +a_2e^{i\theta _2}\vert {10}\rangle -a_3e^{i\theta _3}\vert {11}\rangle$	$\mathbb {1} \otimes \sigma _z$	$a_0e^{i\theta _0}\vert {00}\rangle +a_1e^{i\theta _1}\vert {01}\rangle +a_2e^{i\theta _2}\vert {10}\rangle +a_3e^{i\theta _3}\vert {11}\rangle$
$\vert {\rho ^1}\rangle _{14}$	$\vert {\eta ^3}\rangle _{25}$	$a_0e^{i\theta _0}\vert {00}\rangle -a_1e^{i\theta _1}\vert {01}\rangle -a_2e^{i\theta _2}\vert {10}\rangle +a_3e^{i\theta _3}\vert {11}\rangle$	$\sigma _z \otimes \sigma _z$	$a_0e^{i\theta _0}\vert {00}\rangle +a_1e^{i\theta _1}\vert {01}\rangle +a_2e^{i\theta _2}\vert {10}\rangle +a_3e^{i\theta _3}\vert {11}\rangle$
$\vert {\rho ^1}\rangle _{14}$	$\vert {\eta ^4}\rangle _{25}$	$a_0e^{i\theta _0}\vert {00}\rangle +a_1e^{i\theta _1}\vert {01}\rangle -a_2e^{i\theta _2}\vert {10}\rangle -a_3e^{i\theta _3}\vert {11}\rangle$	$\sigma _z \otimes \mathbb {1}$	$a_0e^{i\theta _0}\vert {00}\rangle +a_1e^{i\theta _1}\vert {01}\rangle +a_2e^{i\theta _2}\vert {10}\rangle +a_3e^{i\theta _3}\vert {11}\rangle$

As shown in Table 1, Caleb can easily obtain the original message by performing simple unitary operations on the state he receives for 4 out of the 16 possible outcomes. This shows that Caleb is able to obtain the original message only 25% of the time, assuming the system is a closed system with no interaction with its environment. However, for an open system that interacts with its environment, it is susceptible to noise, which may affect the entanglement of the qubits under consideration and thus affect the fidelity of the information that reaches Caleb. In the research, we are investigating the fidelity of the information that reaches Caleb, assuming information from Alice and Bob gets to Caleb through a noisy channel that is characterized by SGAD noise.

## Fidelity computation procedure

The final density matrix at Caleb’s side is represented by Eq. (6)

6 $$\begin{aligned} \rho _{out}=Tr_{14,25}(U_0 \xi (\rho ) U_0^\dagger ), \end{aligned}$$

as shown in Eq. (6), $Tr_{14,25}$, represents a partial trace over Bob’s and Alice’s particles, which are particles (1, 4) and (2, 5) for Bob and Alice, respectively. The unitary operator $U_0$ accounts for the sequence of events in the JRSP communication protocol. It is worthy of note that in a perfect JRSP system that is not influenced by noise, the density matrix expressed in Eq. (6) will be very similar to that of the initial state that was transmitted; however, due to the influence of the noisy channel, there is always a notable difference. To investigate the effect of noise on the JRSP protocol, we only consider situations where the JRSP has reached the desired end, as shown in Table 1. In Eq. (6), the value of $U_0$ is then expressed as shown in Eq. (7).

7 $$\begin{aligned} U_0=(I_{14}\otimes I_{25} \otimes \sigma _{36}^k)(I_{14}\otimes |{\phi ^k} \rangle _{25}\langle {\phi ^k} |_{25} \otimes I_{36})(|{\varphi } \rangle _{14}\langle {\varphi } |_{14} \otimes I_{25} \otimes I_{36}), \end{aligned}$$

where $k \epsilon (1,2,3,4)$ and $\sigma _{36}^1=I_1 \otimes I_4$, $\sigma _{36}^2=I_1 \otimes \sigma _Z$ , $\sigma _{36}^3=\sigma _z \otimes \sigma _z$, $\sigma _{36}^4=\sigma _z \otimes I_4$, $U_0$ represent operations that can be carried out by Caleb on his received quantum state. To determine the closeness of the state received by Caleb to the original state shared between Alice and Bob and transmitted, fidelity is used. Since the original state transmitted is given by the Eq. (1), the fidelity of the JRSP system may then be expressed as given in Eq. (8):

8 $$\begin{aligned} F=\langle {T}\vert \rho _{out}\vert {T}\rangle , \end{aligned}$$

When F = 1, it implies a perfect JRSP communication system where the received information is exactly the way it was transmitted, while a value of F less than 1 implies that some transmitted information has been lost in transit. The lower the value of the fidelity, the more information that has been lost.

## Kraus operators, fidelity computation of SGAD channel and associated dissipative channels

The SGAD channel is a dissipative channel that is a generalization of the Amplitude Damping (AD) and Generalized Amplitude Damping (GAD) channels with a squeezing effect. The squeezing effect, which is a quantum asset, delivers added advantages over GAD channels. Therefore, investigating the SGAD channel enables obtaining results about channels involving both non-zero temperatures as well as squeezing parameters^49^. A squeezed reservoir can be devised based on the framework of the installation of a squeezed light field^50^. Experiments investigating the squeezed light atom have been embarked on by Refs.^51,52^. A number of authors agree that a benefit of a squeezed thermal bath is that the rate of degeneration of quantum coherence is decreased, which implies a conservation of quantum resources^53–56^. SGAD has also been proven to alter the progression of the geometric phase of a two-level atomic system^57^. It has also been observed that the SGAD channel has restorative attributes^57,58^.

A number of experiments have been carried out on the SGAD noise model. For example SGAD has been modelled using squeezed light field^50,59^, subthreshold Optical Parametric Oscillator (OPO)^51,52^, beam splitters^60^ and laser cooled trapped ions^61^.

The SGAD channel’s Kraus operator is shown in Eq. (9)^62,63^:

9 $$\begin{aligned} {\left\{ \begin{array}{ll} E_0^{S}= \sqrt{Q} \begin{bmatrix} 1 &{} 0 \\ 0 &{} \sqrt{1-\lambda }\\ \end{bmatrix} \quad E_1^{S}= \sqrt{Q} \begin{bmatrix} 0 &{} \sqrt{\lambda } \\ 0 &{} 0\\ \end{bmatrix} \quad \\ \\ E_2^{S}= \sqrt{1-Q} \begin{bmatrix} \sqrt{1-v} &{} 0 \\ 0 &{} \sqrt{1-\mu }\\ \end{bmatrix} \quad E_3^{S}= \sqrt{1-Q} \begin{bmatrix} 0 &{} \sqrt{\mu }e^{i\Phi } \\ \sqrt{v} &{} 0 \\ \end{bmatrix}, \quad \end{array}\right. } \end{aligned}$$

where the parameters $\mu$, v, and $\lambda$ are as represented in Eqs. (10), (11) and (12) respectively

10 $$\begin{aligned} \mu (t)= & {} \frac{2N+1}{2N(1-Q)} \frac{\sinh ^2\left( \gamma _0 at/2\right) }{\sinh \left( \gamma _0(2N+1)t/2\right) } \exp \left( -\frac{\gamma _0}{2}(2N+1)t\right) , \end{aligned}$$

11 $$\begin{aligned} v(t)= & {} \frac{N}{(1-Q)(2N+1)}\left( 1-\exp \left( -\gamma _0(2N+1)t\right) \right) , \end{aligned}$$

12 $$\begin{aligned} \lambda (t)= & {} \frac{1}{Q}\left( 1-(1-Q)\left( \mu (t)+v(t)\right) -\exp \left( -\gamma _0(2N+1)t\right) \right) , \end{aligned}$$

and $a=\sinh (2r)(2N_{th}+1)$, $N=N_{th}(\cosh ^2(r)+\sinh ^2(r))+\sinh ^2(r)$, $N_{th}={1}/{e^{\left( \frac{\hbar \omega }{k_B T}\right) }-1}$. In order to simplify the notations going forward, we won’t include the time t in the input of any of the equations under the SGAD noise.

To obtain the fidelity for the SGAD channel, first the Kraus operator for the SGAD channel acts on the qubits (1, 4) and (2, 5) corresponding to Alice’s and Bob’s particles, as shown below:

13 $$\begin{aligned} \xi (\rho )_{SGAD}=\sum _{i,j}{(E_i^{S1}\otimes E_i^{S4} \otimes E_j^{S2} \otimes E_j^{S5}) \rho (E_i^{S1}\otimes E_i^{S4} \otimes E_j^{S2} \otimes E_j^{S5})^\dagger }. \end{aligned}$$

**Figure 1:**
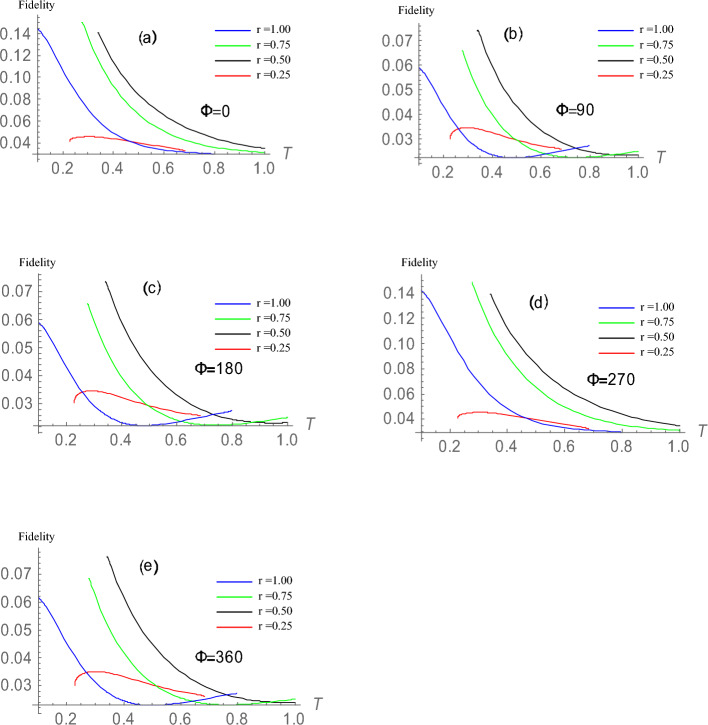
This plot shows the attributes of the SGAD channel. The plots show the variation of fidelity of the SGAD channel at different temperatures and different values of the squeezing parameters r and $\Phi$ as indicated on the plots. When the squeezing parameters r and $\Phi$ are changed while the other parameters remain constant, the fidelity swings high and low around the same value limits. Looking at plots (**b**), (**c**), and (**e**), it is clear that when r = 1 and $\Phi = 90$, 180, and 360, respectively, an increase in fidelity with an increase in temperature is possible. Also, looking at plots (**a**) and (**d**), it is observed that when r = 0.5 and $\Phi =0$ and 270, fidelity is sustained for higher temperatures at which it normally would have been completely diminished. This demonstrates that squeezing parameters can be used to improve fidelity in non-zero temperature thermal baths. To arrive at the plots, the following values were assigned to other SGAD parameters: $\omega =0.5$, Q=0.5, $\gamma =0.5$. where $\mu$, v, and $\lambda$ are functions of temperature T, $\gamma$, $\omega$ and squeezing parameters r as expressed in Eqs. (10), (11) and (12) and units are such that $\hbar \equiv k \equiv 1$.

By substituting the results of Eq. (13) into Eq. (6) and evaluating using Eqs. (7) and (8) we obtain the equation representing the fidelity of the SGAD channel. The equation, however, is quite complex and involves many variables that, due to space constraints, cannot be expressed in this paper. However, the graph expressing the attributes of the SGAD channel is shown in Fig. 1.

### Amplitude damping (AD) Channel

This is one of the channels that can be modeled using the SGAD by assigning some specific values to some of the parameters of the SGAD. The AD channel acts like the interaction of a quantum system with a vacuum bath^49^, It presents the rate of energy loss in a quantum state due to its interaction with a vacuum bath. A lot of work has been carried out on this noise model, and a lot of applications have been found for the model^64–69^. For example, it is used in the basic conceptual structure of the weak Born–Markov approximation in analyzing the spontaneous emission of a photon by a two level system into a photon environment at low temperature. The Kraus operators for the AD channel can be written as^70^:

14 $$\begin{aligned} E_0^{A}= \begin{bmatrix} 1 &{} 0 \\ 0 &{} \sqrt{1-\lambda _{A}}\\ \end{bmatrix}, \ \ \ E_1^{A}= \begin{bmatrix} 0 &{} \sqrt{\lambda _A} \\ 0 &{} 0\\ \end{bmatrix}, \end{aligned}$$

where the decoherence rate $\lambda _A:(0\le \lambda \le 1)$, represents the likelihood of error when particles traverse an AD noisy channel. As declared earlier, AD noise has an effect on only Alice’s and Bob’s qubits, which are (1, 4, 2, 5) Qubits 3 and 6 are not affected by the noisy channel. The effect of the AD channel on the quantum state of the information being transmitted can be represented by the following expression:

15 $$\begin{aligned} \xi (\rho )_A=\sum _{i,j}{(E_i^{A1}\otimes E_i^{A4} \otimes E_j^{A2} \otimes E_j^{A5}) \rho (E_i^{A1}\otimes E_i^{A4} \otimes E_j^{A2} \otimes E_j^{A5})^\dagger }, \end{aligned}$$

where *i* and *j* represent the Kraus operators acting on qubits (1425) that are in superscript in the Eq. (15). To simplify Eq. (14), the noise parameter Q is set to 1, the parameter $\mu$ is set to 0, the parameter v is such that $v=\lambda$ and the squeezing parameters r and $\Phi$ are set to 0. Making these substitutions in Eq. (9) will reduce it to Eq. (14). We obtain the fidelity of the system, which is a measure of how close the final state is to the initial state as:

16 $$\begin{aligned} F_{AD}=\frac{\lambda ^4}{4}-\frac{3 \lambda ^3}{4}+\frac{13 \lambda ^2}{8}-2 \lambda +1. \end{aligned}$$

**Figure 2:**
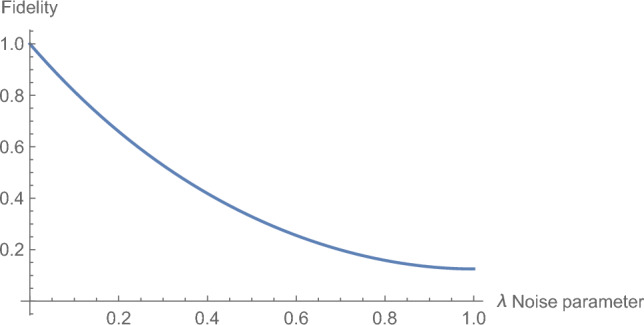
The fidelity of Amplitude Damping model shows decreasing fidelity with an increase in the noise parameter $\lambda _A$. This means that the higher the value of the noise parameter $\lambda _A$ the greater the loss of information along the channel, leading to a higher difference between the information sent by Alice and Bob and the information received by Caleb. The plot clearly shows that the channel’s fidelity is highest, which is 1, when $\lambda _A$ is 0 and lowest, which is ($\frac{1}{8}$), when $\lambda _A$ is 1.

According to Eq. (16), the fidelity of the AD channel is solely determined by the amplitude damping noise parameter ($\lambda _A$). From Fig. 2, it can be seen that when $\lambda _A$ has a maximum value, which is when $\lambda _A=1$, we have the fidelity $F_{AD}=\frac{1}{8}$ which is the minimum fidelity that can be attained in the AD channel, while when $\lambda _A=0$ the fidelity is equal to 1, which signifies a perfect JRSP.

### Generalized amplitude damping channel

This is another channel that may be modeled using the SGAD by assigning specific values to some of its parameters. In a GAD channel, the quantum system loses and gains excitation by interacting with the environment. The GAD channel is utilized in cloning the spontaneous emission of a particle subjected to a vacuum bath with a temperature greater than zero. Equation (17) gives the Kraus operators for the GAD channel^70,71^.

17 $$\begin{aligned} {\left\{ \begin{array}{ll} E_0^{G}= \sqrt{Q} \begin{bmatrix} 1 &{} 0 \\ 0 &{} \sqrt{1-\lambda _{G}}\\ \end{bmatrix}, \quad E_1^{G}= \sqrt{Q} \begin{bmatrix} 0 &{} \sqrt{\lambda _{G}} \\ 0 &{} 0\\ \end{bmatrix}, \quad \\ \\ E_2^{G}= \sqrt{1-Q} \begin{bmatrix} \sqrt{1-\lambda _{G}} &{} 0 \\ 0 &{} 1\\ \end{bmatrix}, \quad E_3^{G}= \sqrt{1-Q} \begin{bmatrix} 0 &{} 0 \\ \sqrt{\lambda _{G}} &{} 0 \\ \end{bmatrix}. \quad \end{array}\right. } \end{aligned}$$

To obtain the GAD channel Kraus operators from Eq. (17), we substitute $\Phi = 0$, $\mu = 0$, and $v = \lambda$. The effect of the GAD channel on the quantum state of the information being transmitted can be represented by the expression given below:

18 $$\begin{aligned} \xi (\rho )_G=\sum _{i,j}{(E_i^{G1}\otimes E_i^{G4} \otimes E_j^{G2} \otimes E_j^{G5}) \rho (E_i^{G1}\otimes E_i^{G4} \otimes E_j^{G2} \otimes E_j^{G5})^\dagger }. \end{aligned}$$

By substituting the result from the computation of Eq. (18) into Eq. (6) and evaluating Eqs. (7) and (8) we obtain the equation for the fidelity of the channel as:

19 $$\begin{aligned} {\left\{ \begin{array}{ll} F_{G}=\frac{\lambda _{G} ^4}{4}-\frac{3 \lambda _{G} ^3}{4}+\frac{13 \lambda _{G} ^2}{8}-2 \lambda _{G} +\frac{\lambda _{G} ^4 Q^4}{2}\\ - \, \frac{3 \lambda _{G} ^3 Q^4}{2}+\frac{11 \lambda _{G} ^2 Q^4}{2}-8 \lambda _{G} Q^4\\ + \, 4 Q^4-\lambda _{G} ^4 Q^3+3 \lambda _{G} ^3 Q^3\\ - \, 11 \lambda _{G} ^2 Q^3+16 \lambda _{G} Q^3\\ - \, 8 Q^3+\frac{3 \lambda _{G}^4 Q^2}{2}-\frac{9 \lambda _{G}^3 Q^2}{2} \\ + \, 12\lambda _{G}^2 Q^2-16 \lambda _{G}Q^2+8Q^2-\lambda _{G} ^4 Q+3 \lambda _{G}^3Q\\ - \, \frac{1/3\lambda _{G} ^2 Q}{2}+8 \lambda _{G}Q-4 Q+1. \end{array}\right. } \end{aligned}$$

**Figure 3:**
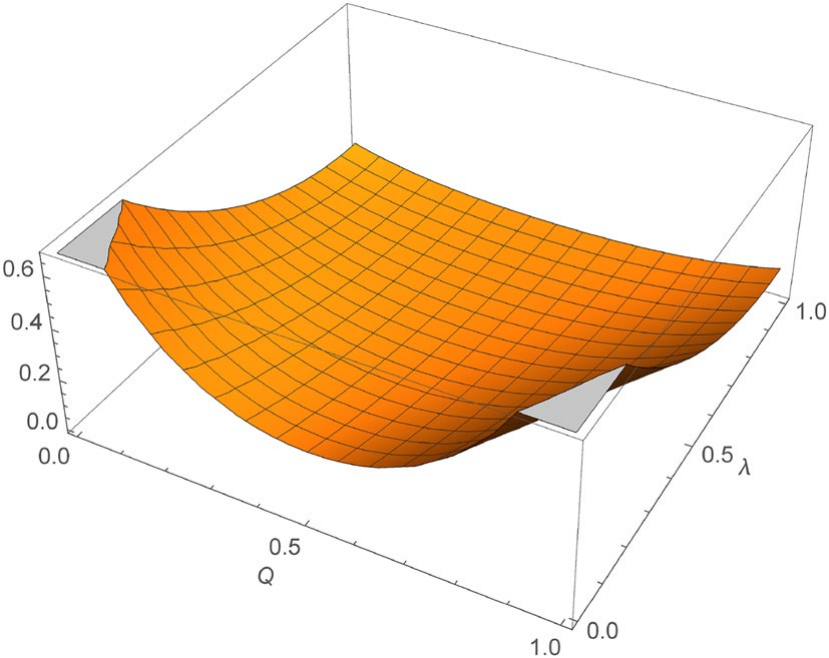
3D plot of fidelity values with varying probability parameter Q and noise parameter $\lambda _G$ in a GAD channel. It can be seen that the fidelity of the JRSP system decreases with increasing values of the probability parameter Q until a value of 0.5, at which point it starts to increase again. The fidelity is maximum at Q = 0 and Q = 1 and minimum at Q = 0.5 when $\lambda _G=0$. At $\lambda _G=1$, the fidelity of the system is $\frac{1}{8}$ regardless of the value of Q, which is the minimum fidelity that can be obtained from the JRSP system. This is very similar to an AD channel. However, it is worthy of note that in a GAD channel, the noise parameter $\lambda _G$ is dependent on temperature.

**Figure 4:**
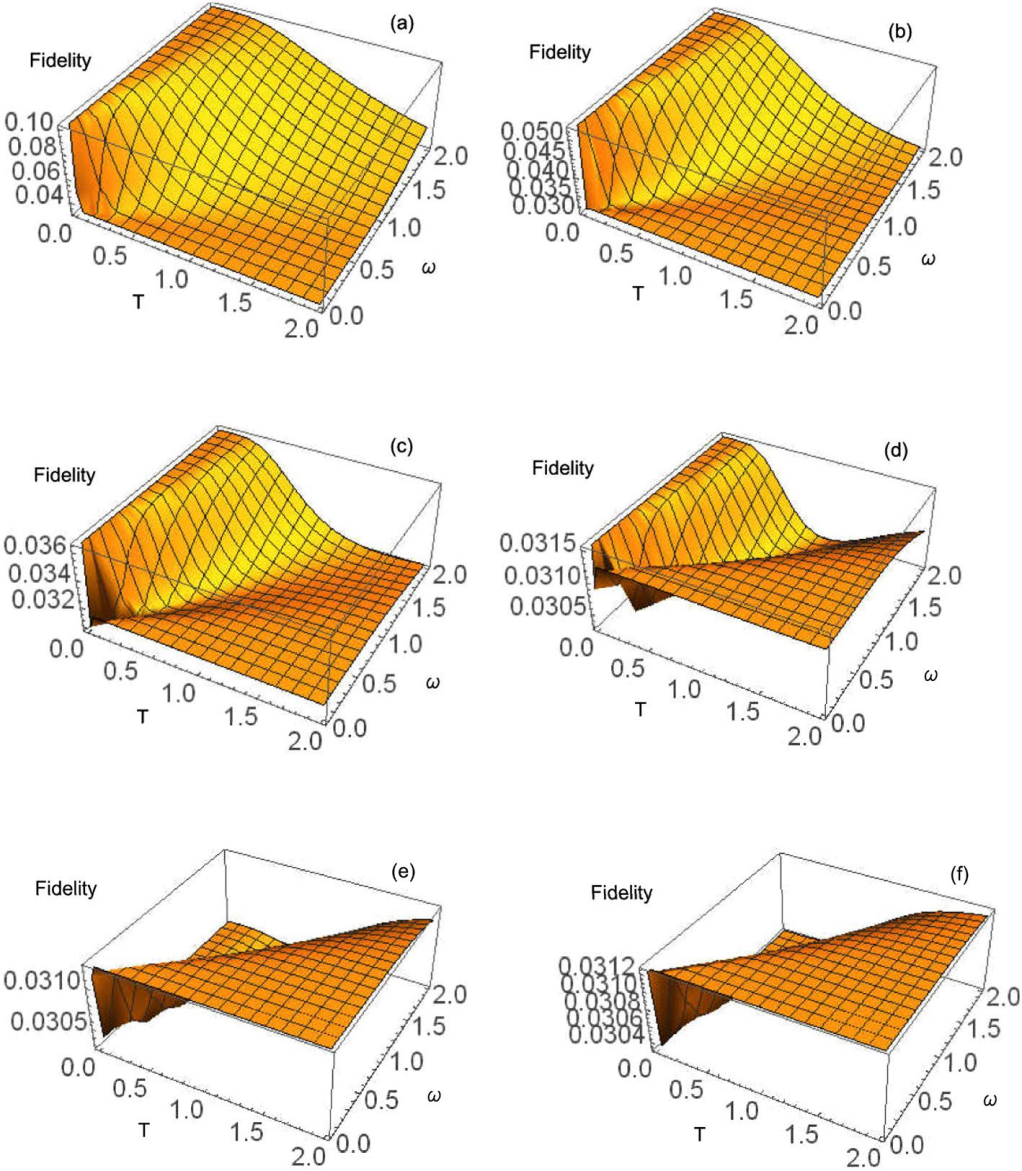
3D plot of variation in fidelity with varying values of temperature and the frequency of the photons, $\omega$. To arrive at these plots, the values of the squeezing parameters r and $\Phi$ were set to 0, $\mu$ was set to 0, and v was set to be equal to $\lambda$ which is equal to $\frac{1}{\exp ^{\frac{\omega }{T}}-1}$ while Q was set to 0.5. The plots a-f shows how fidelity changing with temperature and frequency at spontaneous emission rates $\gamma$ of 0.5, 1.0, 1.5, 2.0, 2.5 and 3.0, respectively in units such that $\hbar \equiv k \equiv 1$. The plots show that increases in temperature and $\omega$ lead to decreases in fidelity; however, the rate of decrease in fidelity with an increase in temperature is higher than the rate of decrease in fidelity with an increase in $\omega$. The plots also show that, generally, the fidelity decreases with an increase in the spontaneous emission rate $\gamma$.

When $Q=0$, Eq. (19) is reduced to $F_{G}=\frac{\lambda ^4}{4}-\frac{3 \lambda ^3}{4}+\frac{13 \lambda ^2}{8}-2 \lambda +1$, which is the fidelity of the AD Channel, when $Q=1$ and $\lambda =0$, Eq. (19) returns a constant value of 1, irrespective of Q which indicates a perfect JRSP. When $Q=1$, Eq. (19) again reduces to $F_{G}=\frac{\lambda ^4}{4}-\frac{3 \lambda ^3}{4}+\frac{13 \lambda ^2}{8}-2 \lambda +1$, which again is the fidelity of an AD channel. When $\lambda _G=1$, Eq. (19) is reduced to $F_G=\frac{Q^4}{2}-Q^3+Q^2-\frac{Q}{2}+\frac{1}{8}$, which always returns $\frac{1}{8}$ regardless of Q. When both Q and $\lambda$ are equal to 1, the fidelity of the channel is $F_G=\frac{1}{8}$ which is it’s minimum fidelity. $\lambda _G$ on the other hand, is temperature-dependent and can be calculated as $\lambda _{G}=1-\exp ^{\gamma _0(2N_{th}+1)}$, where $Q=\frac{N_{th}+1}{2N_{th}+1}$ and $N_{th}= \frac{1}{(\exp ^{\frac{\hbar \omega }{k_B T}-1}-1)}$. With these changes, using the Eq. (19) and $\gamma =0.05$, The expression for the fidelity is obtained, which gives a very complex and long equation that may not be appropriate for this article, but the properties of the fidelity are as shown in Fig. 3. By making $\omega$ constant and varying the temperature and the $\gamma$ parameter, we observe, as shown in Fig. 4) that the fidelity of the given channel decreases with increasing $\gamma$. Taking the values of $\omega$ and Q to be equal to 1, we obtain the equation for the fidelity of the channel to be given by Eq. (20).

20 $$\begin{aligned} F_G=\frac{1}{4} e^{-4 \gamma \left( \frac{2}{e^{\frac{1}{T}}-1}+1\right) }-\frac{1}{4} e^{-3 \gamma \left( \frac{2}{e^{\frac{1}{T}}-1}+1\right) }+\frac{7}{8} e^{-2 \gamma \left( \frac{2}{e^{\frac{1}{T}}-1}+1\right) }+\frac{1}{8}. \end{aligned}$$

## Discussion and conclusion

The fidelities of joint remote preparation of a maximally entangled two qubit state $\vert {B}\rangle =a_0e^{i\theta _0}\vert {00}\rangle +a_1e^{i\theta _1}\vert {01}\rangle +a_2e^{i\theta _2}\vert {10}\rangle +a_3e^{i\theta _3}\vert {11}\rangle$ where $a_0=a_1=a_2=a_3=\frac{1}{2}$ in contact with an SGAD noisy channel, and other dissipative channels, namely the amplitude damping (AD) and the generalized amplitude damping (GAD), have been examined. We found that as the noise parameter $\lambda _A$ increases, the fidelity of the AD channel decreased, with a minimum value of $\frac{1}{8}$ when $\lambda _A=1$ and a maximum value of 1 (which denotes a perfect JRSP) when $\lambda _A=0$. For GAD with a noise parameter $\lambda _G$ where $\lambda _G$ is dependent on temperature T, frequency of photons $\omega$ and spontaneous emission rate $\gamma$, it was observed that fidelity of the channel decreased with increase in temperature, $\omega$ and $\gamma$ still having a value of $\frac{1}{8}$ when $\lambda _G=1$ and having the attributes of a perfect JRSP when $\lambda _G=0$. For the SGAD channel, though it is already established that the fidelity of a JRSP quantum protocol in contact with a thermal bath decreases with increase in temperature, it was observed that it is possible to get an opposing result for particular values of the squeezing parameters r and $\Phi$. When r = 1 and $Phi= 90$, 180, and 360, an increase in fidelity with increasing temperature is observed, while when r = 0.5 and $\Phi =0$ and 270, it is observed that fidelity is sustained at higher temperatures at which it would normally have been diminished.

## Data Availability

This manuscript has no associated data or the data will not be deposited. The data to create the figures would be provided by Cookey Iyen upon request.
